# Supplementary material for: Predicting visual function by interpreting a neuronal wiring diagram
Source: Nature. 2024 Oct 2;634(8032):113–23. doi: 10.1038/s41586-024-07953-5 (PMC11446822; doi:10.1038/s41586-024-07953-5)
Supplement: Supplementary file 6 — CRF and ERF predictions for individual TmY4 and TmY9 cells. Analogous to Supplementary Data 3, but for TmY target types. Shown are the top four monosynaptic pathways, the strongest pathway passing through each of the top ten intermediary types (ranking from Extended Data Fig. 7), and the trisynaptic pathway Tm1–TmY–Dm3–TmY (see the section entitled Prediction of spatial normalization). [file 41586_2024_7953_MOESM6_ESM.zip › DataS4/TmY9qΓèÑ/720575940613089394.pdf]

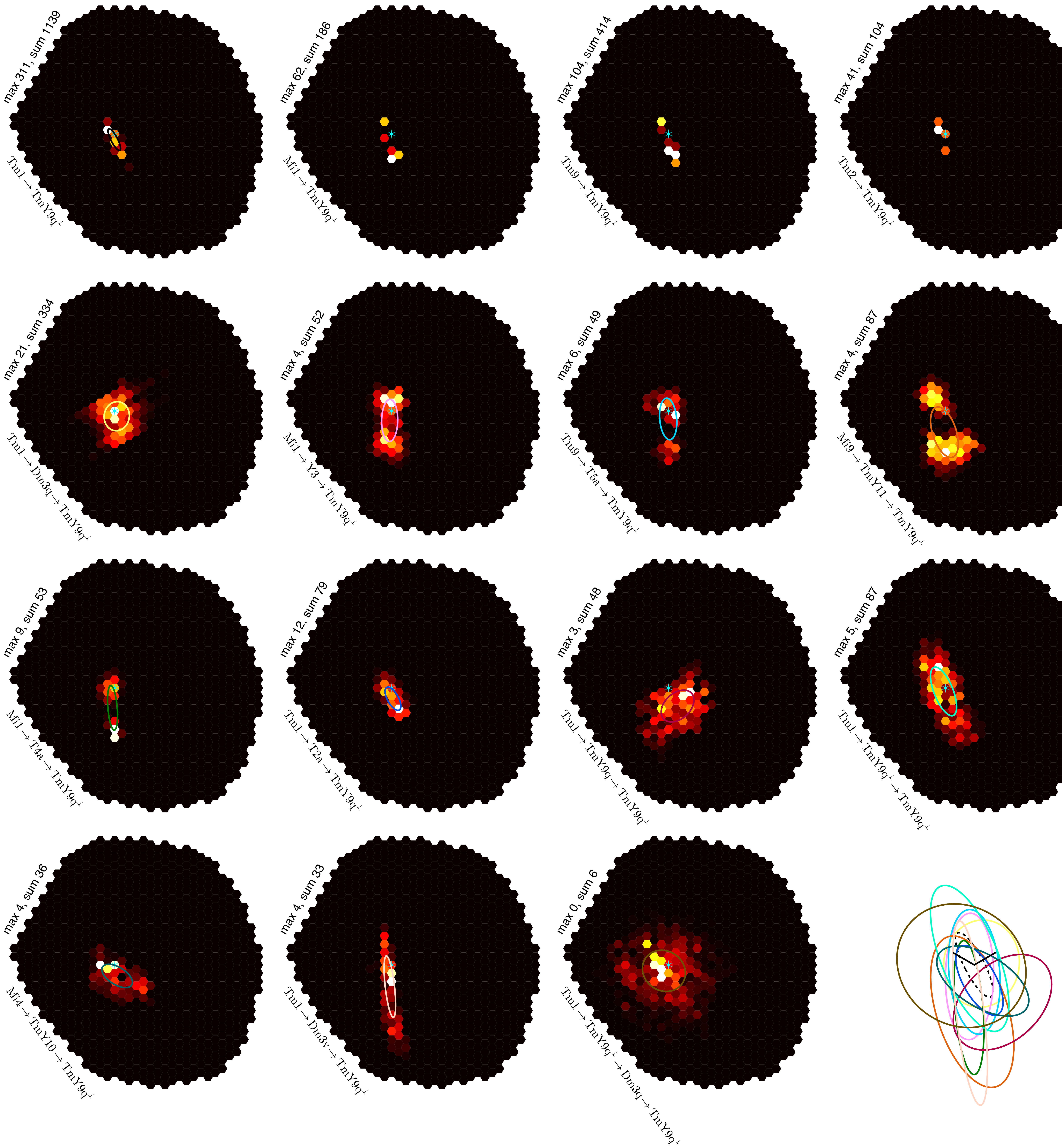
max 311, sum 1139
Tm1 → TmY9q⊥
max 62, sum 186
Mi1 → TmY9q⊥
max 104, sum 414
Tm9 → TmY9q⊥
max 41, sum 104
Tm2 → TmY9q⊥
max 21, sum 334
Tm1 → Dm3q → TmY9q⊥
max 4, sum 52
Mi1 → Y3 → TmY9q⊥
max 6, sum 49
Tm9 → T5a → TmY9q⊥
max 4, sum 87
Mi9 → TmY11 → TmY9q⊥
max 9, sum 53
Mi1 → T4a → TmY9q⊥
max 12, sum 79
Tm1 → T2a → TmY9q⊥
max 3, sum 48
Tm1 → TmY9q → TmY9q⊥
max 5, sum 87
Tm1 → TmY9q⊥ → TmY9q⊥
max 4, sum 36
Mi4 → TmY10 → TmY9q⊥
max 4, sum 33
Tm1 → Dm3v → TmY9q⊥
max 0, sum 6
Tm1 → TmY9q⊥ → Dm3q → TmY9q⊥
